# Innate immunity modulation in the duodenal mucosa induced by REM sleep deprivation during infection with *Trichinella spirallis*

**DOI:** 10.1038/srep45528

**Published:** 2017-04-04

**Authors:** Elizabeth G. Ibarra-Coronado, Armando Pérez-Torres, Ana M. Pantaleón-Martínez, Javier Velazquéz-Moctezuma, Veronica Rodriguez-Mata, Jorge Morales-Montor

**Affiliations:** 1 Departamento de Fisiología, Facultad de Medicina, Universidad Nacional Autónoma de México, México City, México; 2 Departamento de Biología Celular y Tisular, Facultad de Medicina, Universidad Nacional Autónoma de México, México City, México; 3 Departamento de Inmunología, Instituto de Investigaciones Biomédicas, Universidad Nacional Autónoma de México, México City, México; 4 Area de Neurociencias, Departamento de Biología de la Reproducción, CBS, Universidad Autónoma Metropolitana-Iztapalapa, México City, México

## Abstract

Sleep is considered to be an important predictor of the immunity, since the absence of sleep can affect the development of the immune response, and consequently increase the susceptibility to contract an infection. The aim of the present study was to investigate if sleep deprivation and stress induce dysregulation of the duodenal mucous membrane during the acute infection with *Trichinella spiralis*. Our results shows that, in the intestinal mucous membrane, stress and sleep deprivation, produces different effect in the cells, and this effect depends on the studied duodenal compartment, glands or villi. The sleep deprivation affect mast cells mainly, and the stress response is more heterogeneous. Interestingly, in the duodenal mucous membrane, none population of cells in the infected groups responded equally to both conditions. These findings suggest that the response of the intestinal mucous membrane during the infection caused for *T. spiralis* turns out to be affected in the sleep-deprived rats, therefore, the results of the present study sustain the theory that sleep is a fundamental process that is capable of modulating the immune response of mucous membranes, particularly the one generated against the parasite *Trichinella spiralis*.

Sleep is an essential homeostatic process in human beings and other animals. Sleep deprivation, on the other hand, is associated to heart disease^1^, diabetes^2^, obesity^3^, anxiety, depression^4^ and gastrointestinal disorders^5^, and also has a negative influence on the central nervous system and the immune system^6^,^7^, which, in turn, affect the intestinal barrier (IB)^8^. Although most research on sleep deprivation has been focused on its effect over the central nervous system and its functions, the dynamic changes in the IB and the innate mucosal immunity have not gained much attention despite the potential association of gastrointestinal disorders, such as irritable bowel syndrome (IBS) and peptic ulcers, with sleep disorders^9^,^10^.

It is known that the integrity of the gastrointestinal mucosa is kept in part by the immune system^11^ therefore, it also plays a central role in several gastrointestinal disorders including irritable bowel syndrome^12^,^13^, small intestinal bacterial overgrowth and in gastrointestinal parasite establishment and colonization^14^.

Particularry studies suggest that REM sleep deprivation involves changes in the modulation of the immune system and may increase the production of pro-inflammatory cytokines and cells^15^,^16^, which participate in the innate immune response.

While the effects of sleep deprivation on the integrity of the intestinal barrier have been little explored, it has been reported that sleep deprivation leads to the translocation of pathogens into the lymph nodes and other tissues that are not usually colonized by them^17^.

In addition, using confocal endomicroscopy and scanning electron microscopy, it has been recently reported that REM sleep deprivation induce changes in the gastric mucosa showing the initial phases of the acute inflammatory response^18^.

The duodenum is comprised by the four characteristic layers of the gastrointestinal tract: mucosa, submucosa, muscularis externa, and a serosa layer present only on its anterior surface, since the duodenum is largely retroperitoneal and directly adhered to the posterior abdominal wall^19^.

The lamina propria of the duodenum mucosa accomodates numerous migrant and resident cells of the immune system such as eosinophils, neutrophils, mast cells, and macrophages, populating the connective tissue of the villi core, the diffuse lymphatic tissues, and lymphatic nodules. In a healthy person, the eosinophils are located only in the lamina propria, mainly along the villi^20^,^21^.

The enteroendocrine cells, another important component of the enteric tissue, represent a loop between the neuroendocrine system, the central nervous system, and the enteric system, secreting several regulatory hormones of digestive function^22^. The IB is a regulatory interface enabling the selective exchange of nutrients, ions, and water; the function of this barrier is assisted by both endocrine and immune components that modulates absorption and secretion, as well as the presence of pathogenic and commensal agents in the intestinal lumen^23^. As a regulatory interface, the IB is susceptible to modulatory changes caused by hormones, neurotransmitters, and altered states of functional activation, such as an immune response against gastrointestinal parasites^24^.

*Trichinella spiralis* is an intracelular nematode that colonizes the striated muscles of infected mammals; this zoonosis, known as trichinosis, is commonly caused by the consumption of raw or undercooked meat from infected animals^25^. The establishment of the parasite in the duodenum represents the acute, or entherical, phase characterized by goblet cell hyperplasia, increased mucin and intestinal tirefoil factor expression, and an inflammatory infiltration in the lamina propia^26^. At this stage, the intestinal inflammatory infiltrate is comprised of lymphocytes, mast cells, and eosinophils recruited to the intestinal Peyer patches and solitary lymphatic nodes^27^. Mastocytosis in the intestinal mucosa is also a typical feature of infection with *T. spiralis*^27^, such activation of mast cells, followed by the secretion of its mediators, has been involved in the expulsion of parasites^26^.

Despite the extensive research conducted on sleep and immunity, to date, no data has been published regarding whether sleep deprivation affects duodenal integrity in response to gastrointestinal infection with *T. spiralis*. Thus, we sought to analyze the effects of sleep deprivation on the tissue components of the duodenal mucosa and the cell populations involved in immunity and colonization during infection with *T. spiralis*.

## Results

Cell type analysis in the duodenal mucosa. The experimental groups are defined as sleep sufficient (SS), stress, and sleep deprived (SD) in the following description.

### Eosinophils

**Figure 1:**
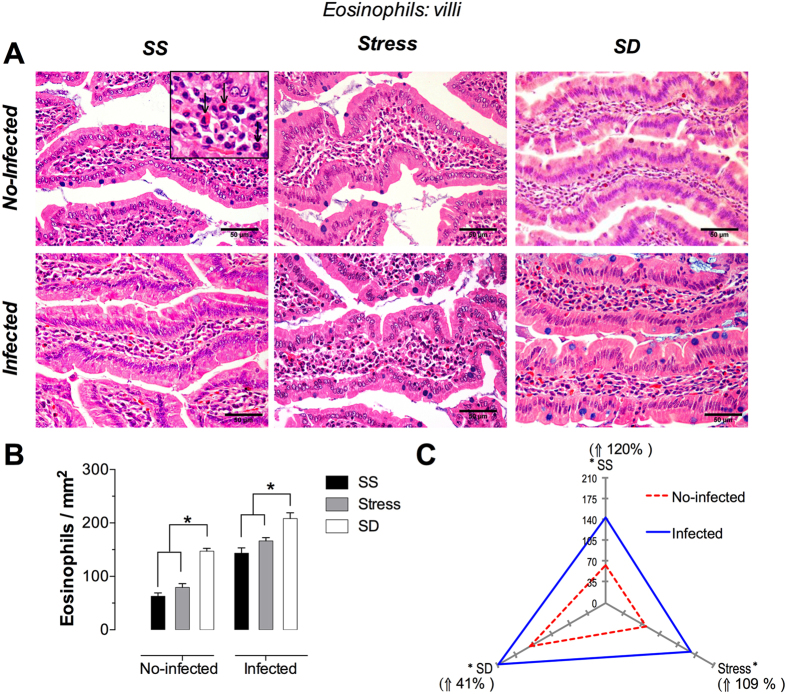
Eosinophils in the duodenal villi. (**A**) Representative photomicrographs of villi in the first portion of duodenum for the condition of sleep sufficient (SS), stress, and sleep deprivation (SD) in no-infected (upper pannel) and infected groups (lower pannel). The square is a magnification of eosinophils (arrow) located in the lamina propria. (**B**) Quantification of eosinophils. SS (solid bars), stress (shaded bars), and SD (open bars) in no-infected and infected groups. (**C**) The differential effect of the infection in the number of eosinophils in SS, stress, and SD in infected (blue continuous line), compared with no-infected (red discontinuous line) groups. The significant differences (*p ≤ 0.05) showed, arise from the comparison of the infected groups versus the no-infected ones. Two way-ANOVA and Bonferroni post-test. *p ≤ 0.05. H&E stain. Scale bars = 50 μm.

**Figure 2:**
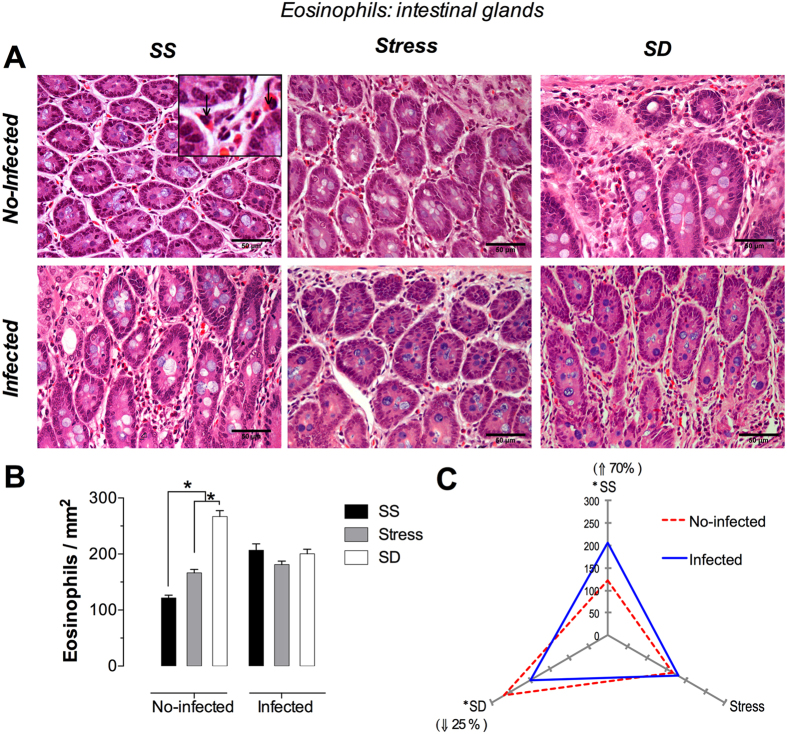
Periglandular eosinophils in the duodenum. (**A**) Representative photomicrographs of periglandular eosinophils in the lamina propria in the first portion of duodenum for the treatments, sleep sufficient (SS), stress, and sleep deprivation (SD), in no-infected (upper pannel) and infected (lower pannel), groups, The square is a magnification of periglandular eosinophils (arrow). (**B**) Quantification of eosinophils in SS (solid bars), stress (shaded bars), and SD (open bars) groups in no-infected and infected groups. (**C**) The differential effect of the infection in the number of periglandular eosinophils in SS, stress, and SD observed in the infected (blue continuous line), compared with no-infected (red discontinuous line) groups. The significant differences (*p ≤ 0.05) showed, arise from the comparison of the infected groups versus the no-infected ones. Two-way ANOVA, and Bonferroni post-test. *p ≤ 0.05. H&E stain. Scale bars = 50 μm.

These cells were identified by the closely packed and specific eosinophilic and refractile granules that virtually fill the cytoplasm (Figs 1 and 2); its most specific morphological feature. In tissue sections, the eosinophil bilobed nucleous was also observed, as in a blood smear, but depending of the cutting plane.

#### Duodenal villi Eosinophils

The top row of photomicrographs shows the no-infected animal groups in the conditions of, from left to right, SS, stress, and SD; whereas the botton row represents the same conditions in the infected animals (Fig. 1A). The duodenal villi in the no-infected groups were lined by normal simple columnar epithelia; however, the stress and SD groups showed a slightly increased number of eosinophils in the lamina propria, whereas an increased number of eosinophils was evident in the lamina propria in the three groups of infected rats. A quantitative analysis (Fig. 1B) revealed that the number of eosinophils is increased in the stress and SD groups (26% and 133%, respectively) when compared with the no-infected SS control group. In the groups of infected rats, the increased magnitude in the number of eosinophils was of 163% in the stress group and of 230% in the sleep deprived groups (SD) in comparison with the no-infected SS control group; however, when compared with the infected, SS group, the increment was of only 16% in the stress group and of 61% in the SD group (Fig. 1C). Moreover, infection per se caused an increased number of eosinophils in SS, stress, and SD conditions estimated in 120%, 109%, and 41%, respectively (Fig. 1C). Note that only SD condition increases the eosinophils number in no-infected groups. However, in infected groups the three conditions presents an increment in the number of eosinophils but was more evident with SD. In fact, the significant increment in eosinophils number was related to SD condition in both no-infected and infected with respect to SS and stress conditions.

#### Periglandular Eosinophils

The morphology of the intestinal glands showed the same type of epithelium as the villi, although with a less abundant periglandular lamina propria continuous with the villus (Fig. 2A). The photomicrographs obtained from the infected groups showed an incremented cellularity in the periglandular lamina propria at the expense of an increased number of eosinophils. The analysis of no-infected rats presented an increased number of eosinophils estimated in 145% and 45% in the SD and stress groups, respectively, when compared with the SS group (Fig. 2B). Surprisingly, the infected rats did not show any change in the number of eosinophils in the stress and SD groups when compared with the SS group (Fig. 2B).

Concerning the effect of *T. spiralis* infection (Fig. 2C), our study found that *T. spiralis* infection in the SS group results in a significant increment of 70% in the number of eosinophils by itself, whereas the stress conditions showed no observable changes (Fig. 2C). Of particular interest, the infected, SD group presented a significant decrement of 25% in the number of eosinophils, contrary to the results observed in the SS control group (Fig. 2C), suggesting that the infection with *T. spiralis* modulates the number of eosinophils produced and further modified by sleep deprivation. Only SD condition in no-infected groups showed a significant increment of eosinophils number with respect to SS and stress conditions. In infected groups, SS, stress, and SD conditions was a not difference in eosinophils number.

### Mucosal mast cells

**Figure 3:**
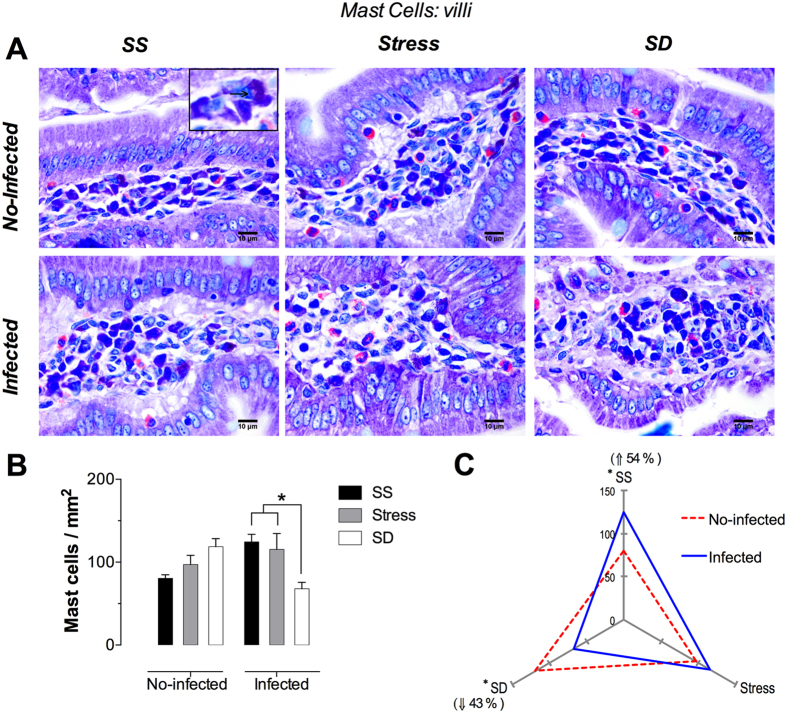
Mast cells in the duodenal villi. (**A**) Representative photomicrographs of mast cells in the villi in the first portion of duodenum for the groups of sleep sufficient (SS), stress, and sleep deprivation (SD) in no-infected (upper pannel) and infected (lower pannel) groups. The square is a magnification of mast cells in lamina propria. (**B**) Quantification of mast cells for SS (solid bars), stress (shaded bars), and SD (open bars) conditions in no-infected and infected groups (**C**) The differential effect of the infection in the number of villi mast cells in SS, stress, and SD observed in infected (blue continuous line), compared with no-infected (red discontinuous line) groups. Two-way ANOVA, and Bonferroni post-test. *p ≤ 0.05. Giemsa stain. Scale bars = 50 μm.

**Figure 4:**
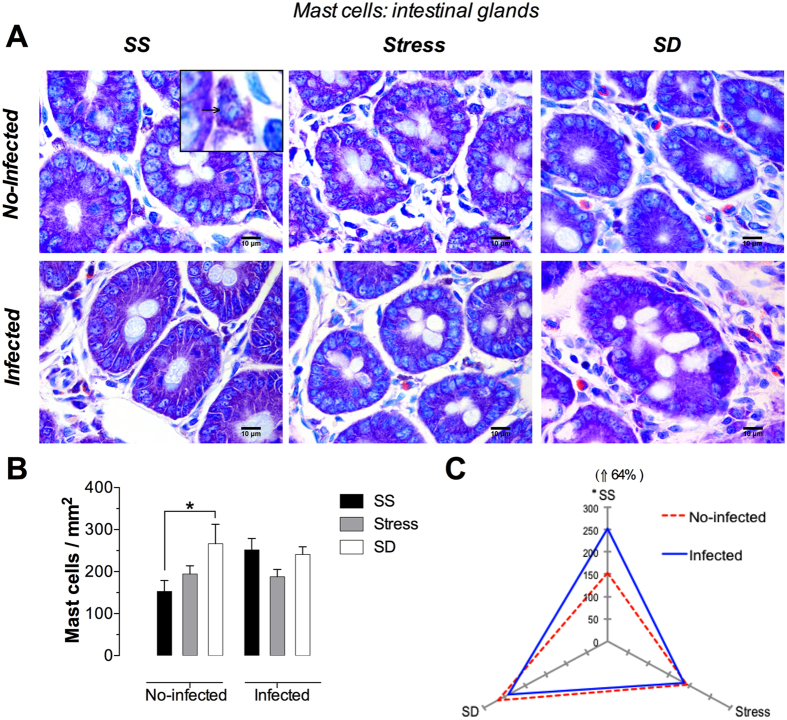
Periglandular mast cells in the duodenum. (**A**) Representative photomicrographs of periglandular mast cells in lamina propria in the first portion of the duodenum for different treatments, sleep sufficient (SS), stress, and sleep deprivation (SD) in no-infected (upper pannel) and infected (lower pannel) groups. The square is a magnification of a single periglandular mast cell. (**B**) Quantification of mast cells in the different treatments. SS (solid bars), stress (shaded bars), and SD (open bars) in no-infected and infected groups. (**C**) The distinguishing effect of the infection in the number of mast cells in SS, stress, and SD observed in infected (blue continuous line), compared with no-infected (red discontinuous line) groups. Two-way ANOVA, and Bonferroni post-test. *p ≤ 0.05. Giemsa stain. Scale bars = 50 μm.

These cells appeared as ovoid, but also showed a polyhedral or fusiform shape with long and slender cytoplasm processes, with a spherical nucleus surrounded by numerous, homogeneous and intensely basophilic or metacromathic granules (Figs 3 and 4, insert). These morphological characteristics facilitated the identification and analysis of mast cells in the lamina propria.

#### Duodenal Villi Mast Cells

The identification and quantification of mast cells was performed using Giemsa staining (Fig. 3). As in the case of eosinophils, no damage was observed in the mucosa layer covering the villi of the no-infected groups, although in the infected, SS and stress groups, a thickened lamina propria could be observed.

The quantification analysis of mast cells revealed that the no-infected animals presented and increased number (without significant) of these cells in both stress and sleep deprivation conditions of 20 and 47%, respectively (Fig. 3B). On the other hand, the infected groups showed an increased number of mast cells in the infected SS and stress groups of 154% and 143% versus the no-infected SS group; however, the infected SD groups showed a significant decrement of ~40% versus the infected SS and stress groups (3B). Interestingly, under SS conditions the infection with *T. spiralis* causes a significant increament of 54% in the number of mast cells, whereas under SD conditions the infection caused a significant decrement of 43%; regardless, the infection with *T. spiralis* did not provoke significant any changes under stress conditions (Fig. 3C). None of the three conditions of the no-infected groups showed significant changes in the number of mast cells. Only the SD condition in infected groups showed a significant decrease in these cells with respect to SS and stress conditions. Note that the infection (blue continuous line) induced a significant increase of villi mast cells in SS condition, while in SD induced a significant decrease with respect to no-infected groups (red discontinuous line).

#### Periglandular Mast Cells

The analyzed mast cells showed a typical morphology in the periglandular area, which is similar to amorphous and granular cells (Fig. 4A). In the no-infected groups, remarkable changes were observed in the SD groups, in which an increased number of these cells was evident (Fig. 4A). The microphotographs corresponding to the infected groups showed a homogeneous distribution of these cells interacting with eosinophils (Fig. 4A). The quantitative analysis in the no-infected groups showed that SD conditions produces a significant increment of 74% whereas a slight, non-significant increment was observed in the stress groups when compared against the no-infected SS groups (Fig. 4B). The infected groups under stress or SD conditions did not show any significant changes compared with the infected, SS groups. Consequently, we observed that the increment caused by SD conditions in the no-infected animals was attenuated by the infection with *T. spiralis* (Fig. 4B). In contrast, we observed that infection alone had a clear effect under SS conditions, as this group showed a significant increment of 64% whereas in SD conditions we observed a slight, non-significant decrement of 10% (Fig. 4C). In no-infected groups, only SD condition showed a discrete buy significant increment of mast cells. The infected groups did not show differences with respect to the number of periglandular mast cells among SS, stress and SD conditions. Infection causes a change only in SS condition compared to no-infected groups.

### Enteroendocrine cells

**Figure 5:**
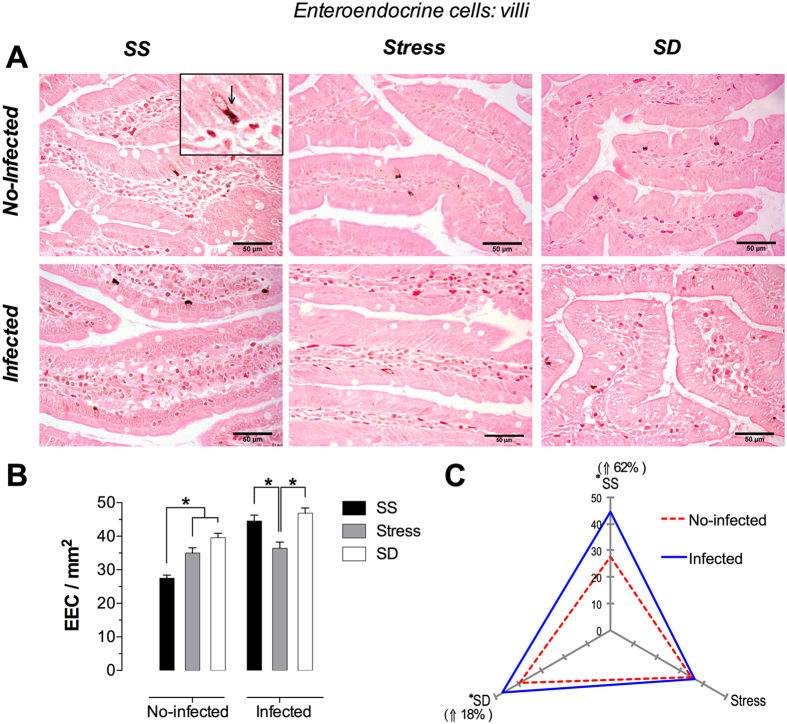
Enteroendocrine cells in the duodenal villi. (**A**) Representative photomicrographs of villi in the first portion of duodenum for the different treatments of sleep sufficient (SS), stress, and sleep deprivation (SD) in no-infected (upper pannel) and infected (lower pannel) groups. The square is a magnification of typical intraepithelial enteroendocrine cell (arrow). (**B**) Quantification of villi enteroendocrine cells in SS (solid bars), stress (shaded bars), and SD (open bars) in no-infected and infected groups. (**C**) The differential effect of the infection in the number of villi enteroendocrine cells in SS, stress, and SD observed in infected (blue continuous line), compared with no-infected (red discontinuous line) groups. Two-way ANOVA, and Bonferroni post-test. *p ≤ 0.05. Fontana Masson stain. Scale bars = 50 μm.

These cells were identified inside of villi and intestinal gland epithelium, confined to the vicinity of the basal lamina. They showed a pyramidal shape, with a narrow vertex extending towards the apical surface of intestinal glands and villi but, apparently, does not reach the lumen. Its wide cell base is adjacent to the basal lamina. The basal cytoplasm, and to a lesser extent the apical cytoplasm, presented argentaffin granules that give the enteroendocrine cells blackened or dark brown color, which easily distinguished from other epithelial cells (Fig. 5).

#### Duodenal villi Enteroendocrine cells

The histological analysis corresponding to the infected SS and SD groups showed a slight increment in the number of enteroendocrine cells (Fig. 5A); however, the cell number in the no-infected animals was significantly increased under SD and stress conditions (43 and 27%, respectively) (Fig. 5B). In the infected groups, stress conditions resulted in a significant decrement of ~15% when compared with the SS and SD infected groups (Fig. 5B). Furthermore, infection with *T. spiralis* resulted in a significantly increased number of these cells under SS and SD conditions (62% and 18%, respectively) (Fig. 5C). The stress and SD conditions of no-infected groups had a significant increment in enteroendocrine cells with respect to SS condition. In infected groups, only stress condition showed a significant decrease in these cells in comparition to SS and SD condition. Note that infection in SS and SD conditions provoked a significant increase of enteroendocrine cells with respect to no-infected groups.

#### Glandular Enteroendocrine Cells

**Figure 6:**
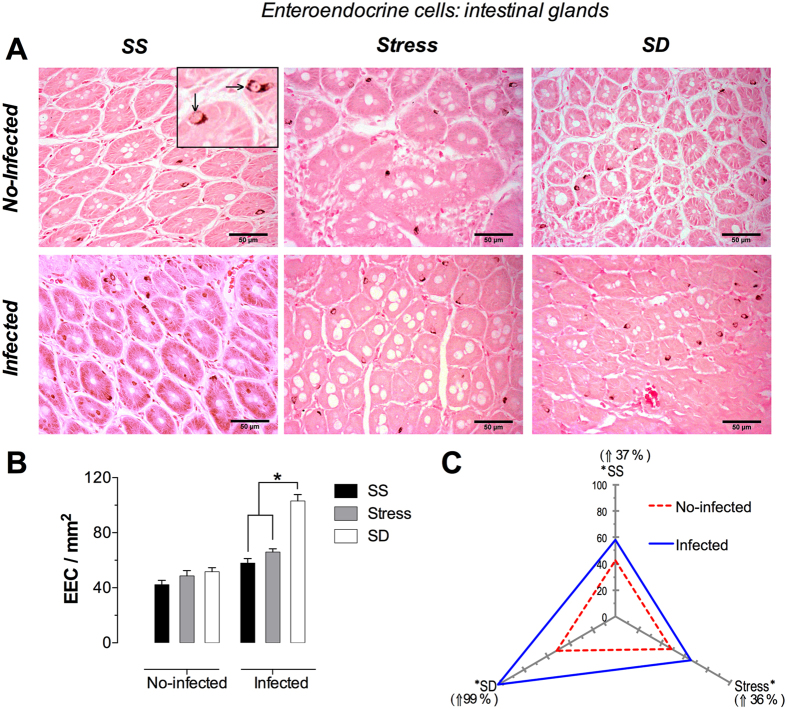
Glandular enteroendocrine cells in the duodenum. (**A**) Representative photomicrographs of enteroendocrine cells in glands in the first portion of duodenum of the diiferent treatments, sleep sufficient (SS), stress, and sleep deprivation (SD) in no-infected (upper pannel) and infected (lower pannel) groups. The square is a magnification of duodenal glands with some enteroendocrine cells. (**B**) Quantification of enteroendocrine cells for SS (solid bars), stress (shaded bars), and SD (open bars) in no-infected and infected groups. (**C**) The differential effect of the infection in the number of enteroendocrine cells in SS, stress, and SD in infected (blue continuous line), compared with no-infected (red discontinuous line) groups. Two-way ANOVA, and Bonferroni post-test. *p ≤ 0.05. Fontana Masson stain. Scale bars = 50 μm.

Enteroendocrine cells could be identified inside the glands, cytoplasm facing towards the exterior, with a color and texture similar to the villi (Fig. 6). The photomicrographic analysis of the no-infected groups showed a homogeneous distribution of these cells in all the tested conditions, whereas the infected groups showed a clear increment in the number of these cells under SD. Although the no-infected animals showed no change under any of the tested conditions, the infected animals did exhibit a significantly increased cell number of 78% under SD conditions when compared with the SS infected group (Fig. 6B). On the other hand, we observed that infection with *T. spiralis* resulted in a modified cell number in every tested condition, where SS and stress presented a slight but significant increment of 37 and 36%, respectively; on the other hand, SD conditions resulted in a clear increment of around 99% (Fig. 6C). In all conditions of no-infected groups no changes in number of enteroendocrine cells was observed; however, in infected groups, the SD condition showed a significant increment of enteroendocrine cells compared to SS and stress conditions.

### Goblet cells

**Figure 7:**
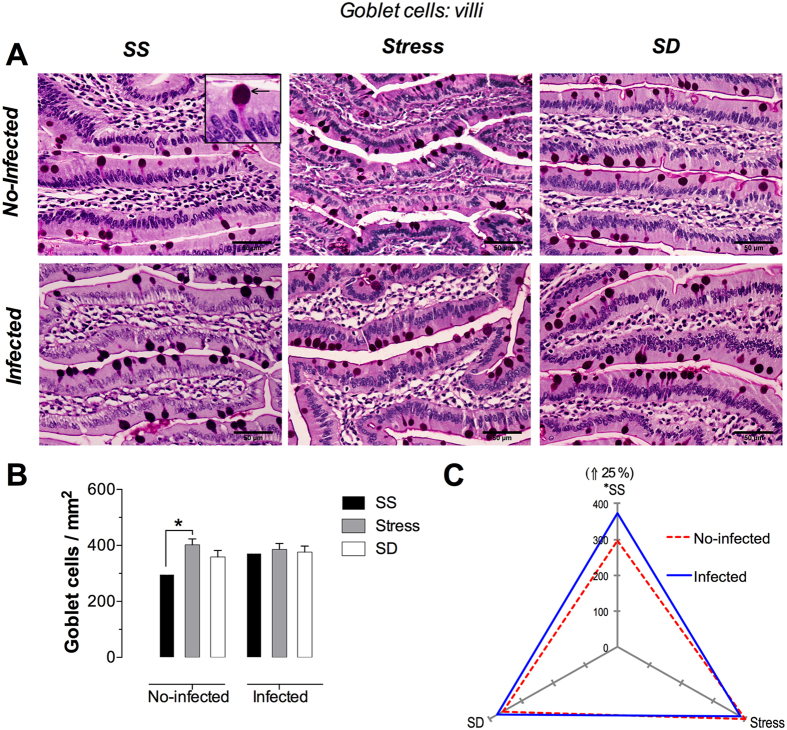
Goblet cells in the duodenal villi. (**A**) Representative photomicrographs of globet cells in villi in the first portion of duodenum for the different treatments of sleep sufficient (SS), stress, and sleep deprivation (SD) in no-infected (upper pannel) and infected (lower pannel) groups. The square is a magnification of typical PAS-positive goblet cell. (**B**) The quantification of goblet cells in SS (solid bars), stress (shaded bars), and SD (open bars) treatments in no-infected and infected groups. (**C**) The differential effect of the infection in the number of goblet cells in SS, stress, and SD observed in the infected (blue continuous line), compared with no-infected (red discontinuous line) groups. Two-way ANOVA, and Bonferroni post-test. *p ≤ 0.05. PAS procedure. Scale bars = 50 μm.

**Figure 8:**
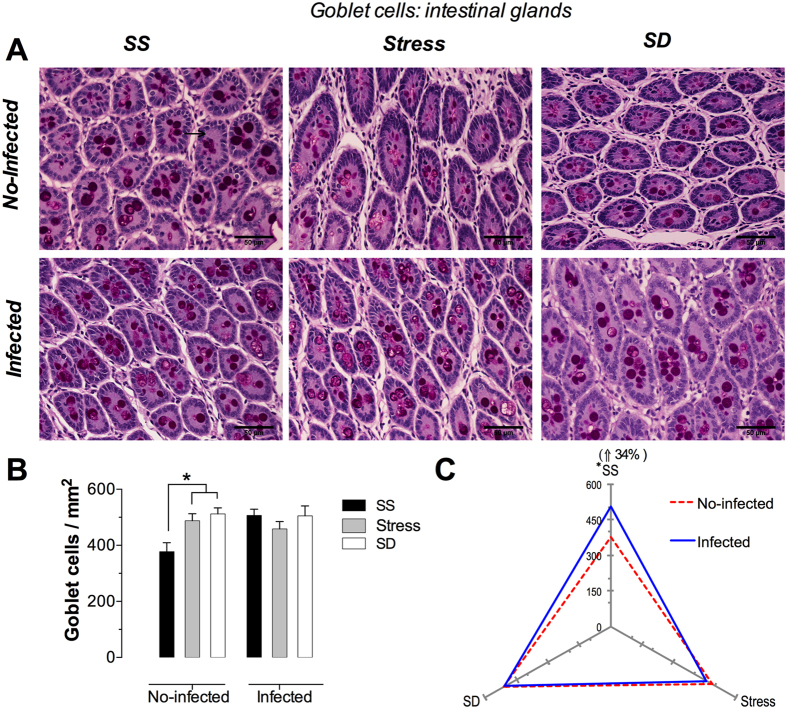
Glandular goblet cells in the duodenum. (**A**) Representative photomicrographs of duodenal glands in the first portion of duodenum for the condition of sleep sufficient (SS), stress, and sleep deprivation (SD) in no-infected (upper pannel) and infected (lower pannel) groups. The insert is a magnification of duodenal glands with three PAS-positive enteroendocrine cells. (**B**) Quantification of glandular goblet cells for SS (solid bars), stress (shaded bars), and SD (open bars) treatments in no-infected and infected groups. (**C**) The differential effect of the infection in the number of goblet cells in SS, stress, and SD conditions observed in infected group (blue continuous line), compared with no-infected (red discontinuous line) groups. Two-way ANOVA, and Bonferroni post-test. *p ≤ 0.05. PAS procedure. Scale bars = 50 μm.

These cells were identified as PAS-positive columnar cells scattered among enterocytes of intestinal glands and villi epithelium. Additionally, and even more distinctive morphologically, goblet cells were easily identified by their cup-shaped apical domain containing PAS-positive mucus granules, a by their narrow basal domain that attach it to the basal lamina (Figs 7 and 8, insert).

#### Duodenal villi Goblet cells

The microphotography analysis shows the homogeneous distribution of these cells along the villi, although no change in cell number could be observed under any of the tested conditions (Fig. 7A). The no-infected groups presented a significant increment of 35% under stress conditions only, whereas the infected groups did not show significant changes under stress or SD conditions when compared with the infected SS group (Fig. 7B). When analyzing the infection effect of *T. spiralis* in each of the tested conditions, it was apparent that it causes a slight, although significant, change of 25% under SS conditions only whereas no effect was observed under the remaining conditions (Fig. 7C). The stress condition in no-infected groups had a significant increase of goblet cells with respect to SS condition but in the three conditions of infected groups no changes in these cells was observed. The infection (blue continuous line) produced a significant increment of goblet cells only in SS condition, compared to three conditions of no-infected groups.

#### Glands Goblet cells

Goblet cells present an intense pink color when fully loaded with granules or a slight pink shade, with a clear center, when the cells have secreted their content into the microenvironment (Fig. 8). The micrography analysis of the infected groups (Fig. 8A) shows an increased number of goblet cells under stress and SD conditions. Moreover, the composition of these cells also is different, as loaded goblet cells are predominant under SS conditions whereas under stress and SD conditions most goblet cells are empty (secretory cells), evidenced by the presence of empty vacuoles (in white). The quantification analysis of goblet cells in the no-infected groups under stress and SD conditions shows a significantly increased number of cells estimated in 29% and 36%, respectively; whereas the analysis of the infected groups did not show any significant differences in any of the tested conditions (Fig. 8B). The infection effect of *T. spiralis* resulted in a significantly increased of 34% in the number of goblet cells in the SS groups only (Fig. 8C). Stress and SD conditions of no-infected groups showed a significant increment of goblet cells number, while all conditions of infected groups no changes were observed in these cells. The infection produced a significant increase of glandular goblet cells only for SS condition of infected group, compared to no-infected groups.

## Discussion

Sleep deprivation is a common occurrence in modern society^28^ resulting from a variety of factors such as social and physiological stress, and environmental insults, such as infections and pollutants^29^. Because sleep is an essential homeostatic process for animals, sleep deprivation has a severe negative influence on all systems of the body^30^. Studies on the experimental manipulation of sleep have focused on the effects of sleep deprivation over the neural functions and blood flow of the brain^31^,^32^; however, the dynamic changes of the intestinal barrier due to sleep deprivation have not gained much attention. In a previous study by our group, we evaluated whether sleep deprivation causes the dysregulation of immune variables during an immune response against the helminth parasite *T. spiralis*^33^. Since sleep deprivation is a form of stress per se, in that study we compared stress alone (consisting in movement restriction and single housing) with sleep deprivation in both control and infected rats. Interestingly, the results obtained demonstrated that sleep deprivation and stress doesn’t produces differences in corticosterone levels at three conditions, sleep, stress and sleep deprivation. However, an increase was observed in the group of animals that were only infected^33^. We shown that even though sleep deprivation can be categorized as a type of stress, the main component of this is sleep loss, and the two stimuli have a differential effect in two compartments of the immune system, namely spleen and mesenteric lymph nodes; also, the populations responded different. We showed that the population cells responded different. We find tha natural killer cells (NK+) are most responsive to sleep deprivation, and the stress response is more heterogeneous. However, in that study we did not evaluate other mechanisms of host defence^33^.

As a regulatory interface, the duodenal barrier is susceptible to modulatory changes caused by hormones, neurotransmitters, and altered states of immune activation. Homeostatic regulation occurs during acute sleep deprivation and impaired in chronic sleep disorders, such as chronic sleep apnea^30^,^34^.

The analysis of the cellular subpopulations was performed at 48 h of infection, because the analysis corresponds to the stage of establishment of the infection, and therefore the mechanisms to expel the parasite have not been established, in our previous study we observed that the parasite load did not show differentiation between the groups^33^. As for the analysis realized in the intestinal mucous membrane, we demonstrated that the conditions of deprivation of sleep (SD) and stress have a distinguishing modulating effect, which depends on the cellular type and on its location.

In the no-infected groups, it was observed that the SD condition presented significant changes in the number of eosinophils and of mast cells. In both cellular types a significant increase was observed in the periglandular area with respect to the group no-infected sufficient sleep (SS), while at villi level, the SD produced an alone significant increase in the number of eosinophils. Our results demonstrate that these cellular types are more sensitive to the deprivation of sleep, and that this condition provokes an inflammatory process both in villi and in the area periglandular. Furthermore, in no-infected groups, the condition of stress, induced a significant increase in the number of caliciform cells at villi level, while the condition of SD produce an increase of glandular goblet cells, in addition, an increase in the number of enteroendocrine cells in villi is seen. The increase in the number of eosinophils observed in the condition of SD was significantly higger than the observed one in the condition of stress.

Sleep deprivation also affected different cell populations in the villi of the infected animals provoking a significantly increased number of eosinophils, whereas mast cell presence was significantly diminished when compared with the infected SS group. This observation becomes interesting since these two cell types are closely related and induce a reciprocal recruiting. Nevertheless, in the present study we observe that while the number of eosinophils was increased, the number of mast cells was diminished which could be due to the cells being degranulated and are in the process of reloading granules, as described in some cases of airways allergies. At glandular level, a significantly increased number of enteroendocrine cells under SD conditions were observed when compared with the infected SS group. The infected group under stress conditions showed a significant decrement in the number of enteroendocrine cells in the villi when compared with the infected SS group. Since the enteroendocrine cells play a fundamental role between the immune system and the enteric nervous system during a severe infectious process, this cell population could be essential in the development of an immune response. The stress and SD conditions exert a modulating effect on the epithelium, and the numerical and functional modification of both enteroendocrine and goblet cells might alter the dynamic integrity of the mucosal barrier protecting the host from infection and potentially inflammatory stimuli. In agreement with the previously stated, Everson and Toth^17^ reported that SD could induce a bacterial translocation towards diverse tissues normally lacking these bacteria. Presumably, this could also explain the observed interaction between inflammation and eosinophilia in the injured gastric mucosa after SD. The study of sleep deprivation needs the simulation of the actual physiological conditions, such as an antigenic challenge, without excluding the interaction of other systems responding to the same stimuli. Recently, using impedance spectroscopy and confocal endomicroscopy we have reported that after 48 hrs of sleep deprivation, gastric mucosa shows signs of epithelial loss and after 96 hrs mucosal epithelium shows isquemic changes^35^. As it has been reported^36^, there is a total loss of REM sleep during the four days of deprivation, as well as a significant decrease of REM sleep in the large platform group. Thus, the observed effect are clearly due to the total suppression of REM sleep but the stress component of the technique and the participation of slow wave sleep loss remains to be elucidated.

**Figure 9:**
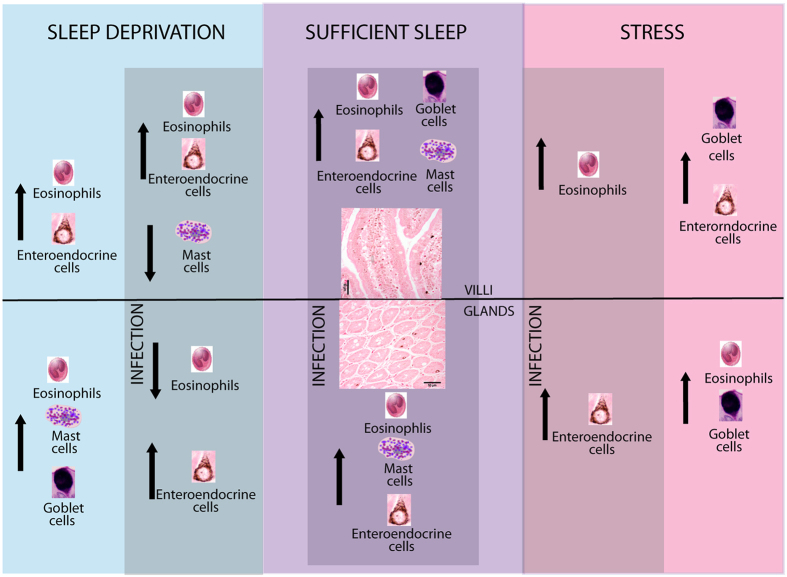
Summary of all changes in the number of some cells present in the intestinal mucosa of the rat duodenum, related to innate immunity. Analysis include *Trichinella spiralis* infected and no-infected groups subjected to sufficient sleep (SS), stress and sleep deprivation (SD). Arrows indicate the increase or decrease in cell types located in intestinal villi or in intestinal glands.

Figure 9 depicts a summary of all the results, and included all changes in the number of the analyzed cells related to innate immunity that are present in the intestinal mucosa of the rat duodenum. The figure include all the analysis of *Trichinella spiralis* infected and no-infected groups, that were subjected to sufficient sleep (SS), stress and sleep deprivation (SD). Arrows indicate the increase or decrease in cell types located in intestinal villi or in intestinal glands.

Considering the complexity involved in the study of such interactions between the immune, nervous, and endocrine systems, as well as their different response to the same stimuli, the present study is an effort in the analysis of the different conditions modifying these interactions. To our knowledge, this is the first study reporting the effects of sleep deprivation and stress on the histological changes and cell dynamics in the duodenal mucosa in response to a gastroenteric *T. spiralis* infection.

## Material and Methods

### Ethics Statement

The protocol used in this study was approved by The Committee on Ethics in Animal Experimentation of the UAM-Iztapalapa. The respective Committee on Care and Use of Laboratory Animals constantly evaluated animal care and experimental procedures at the Universidad Autonoma Metropolitana (UAM), adhering to the official Mexican regulations (NOM-062-ZOO-1999). Mexican regulations are in strict accordance with the recommendations included in the Guide for the Care and Use of Laboratory Animals of the National Institute of Health (NIH) to ensure compliance with the established international regulations and guidelines.

### Animals and experimental groups

Male Wistar rats were used in this study (200 ± 10 g weight), the animals were single housed in a room with controlled temperature (22–24 °C) and 12-hour light-dark conditions. The diet consisted of Harlan 2018 and sterile water ad libitum.

### Experimental Groups

The animals were organized into six groups of 7 animals each; the experimental conditions were as follows:

     No-Infected Animals

(1)  Sleep Suficient (SS): rats were kept in their home cage, uninfected and with undisturbed sleep.

(2)  Stress (S): rats were placed in similar condition as the sleep-deprived group but the size of the platform was large enough to allow them have both slow wave and REM sleep.

(3)  Sleep Deprived (SD): rats were placed in the sleep deprivation condition described above.Infected Animals

     Infected Animals

(4)  Sleep Suficient (SS): rats were infected with *T. spiralis* as described above.

(5)  Stress (S): Animals were infected with *T. spiralis* and placed in large-platform stress conditions.

(6)  Sleep Deprived (SD): rats were sleep deprived and infected after 24 hors of sleep deprivation as described in the methods above.

### Experimental procedure

Since our objective was to evaluate the initial immune response that occurs during infection with *T. spiralis*, at the time of infection the histological analysis was performed 48 h. So the experimental protocol for sleep deprived (SD) infected group included an initial 24 h of sleep deprivation beginning at light phase, after which the rats were infected with 1500 muscle larvae (ML) of *Trichinella spiralis*. The deprivation procedure continued for additional 48 h to prevent sleep rebound. So the total time of deprivation was 72 h and the infection time was 48 h. The total time of deprivation sleep was 72 h, a time less than the time in which the presence of skin lessions^37^.

The protocol for the group stress infected consisted of an initial period of 24 h of stress; at this time the rats were infected. The stress procedure followed for 48 h. For this group the total time of stress was 72 h and the infection time was 48 h. The infected group consisted of infected rats with a time of 48 h, which are infected at the same time that groups SD infected and stress infected. The group SD, was deprivated for 72 h, at the same time that the SD infected group, and the stress group was stressed for 72 h at the same time that stress infected group. The sleep sufficient group wasn’t infected or deprived, the rats were always housing singly in home cages.

### Sleep deprivation

Sleep deprivation was achieved using the island technique, slightly modified from the original reported by Jouvet *et al*.^36^. This procedure is based on the occurrence of muscle atonia during REM sleep. In brief, the rats were placed in rectangular cages with a small central cylindrical platform (4.5 cm diameter, 5 cm high) surrounded by water (2–2.5 cm from the bottom of the cage). In this condition, the rats spent most of the time on the platform. They are awake and they even can reach slow wave sleep stage. Every time that rat reaches REM sleep, fall into the water and wake up. This method suppresses completely REM sleep and induces a slight decrease of slow wave sleep. The water in the cage was changed daily.

As we want to explore the acute effects of REM deprivation, rats were kept in this condition for 96 hrs. Procedure starts at the beginning of the light period and finishing at the end of the dark period four days later. Previous reports indicate that more than 4 days of REM sleep deprivation induce a generalized physiological breakdown^37^. To reduce the stress component of the original procedure, it was slightly modified reducing the height of the platform and the water level. Thus, the rats can get down from the platform and freely walk around the cage, reducing the immobilization stress. Food and water were available ad libbitum.

### Stress Control

Since the early reports of Jouvet *et al*., Cohen *et al*., Mendelson *et al*.^38^,^39^,^40^, some procedures have been proposed as a suitable control group for REM deprived animals. The use of a large platform instead of the small platform was proposed as an adequate control for the stressful situation inherent to the original technique. In addition, the large platform allows the rats to reach REM sleep without falling into the water. This procedure induces only a slight decrease of both REM and slow wave sleep^36^. Thus, a large platform stress control group was used. In this group, animals were kept in exactly the same conditions as the REM deprived group and the only difference is the size of the platform (16 cm diameter) that allows the rats to reach REM sleep stage.

### Infection procedure

The infection procedure was performed as previously described^33^. Briefly, consisted in inserting a gastric catheter, injecting 1500 muscle larvae of *T. spiralis* suspended in 500 μl of 1x PBS directly into the upper stomach. At the end of the experimental protocol, the rats were euthanized and adult worms recovered by dissecting the small intestine into small sections, washed twice in 1X PBS, and incubated in sterile 1X PBS for 3 h at 37 °C. Following incubation, the sedimented parasites were collected, washed in 1X PBS and quantified under a stereoscopic microscope.

### Histological analysis

The duodenum samples obtained from the animals were fixed in 4% paraformaldehyde (J.T. Baker, México), dehydrated, and embedded in paraffin. Non-serial, longitudinal tissue blocks were cut into 4 μm thick sections and mounted on poly-L-lysine coated slides (Sigma, St Louis, MO, USA). The histological anlyses were performed with hematoxylin- eosin staining to identify eosinophils, Giemsa stain for mucosal mast cells, periodic acid Schiff procedure (PAS) for goblet cells, and Fontana-Masson method for enteroendocrine cells. The number of each cell type on the intestinal glands and villi was calculated using a 40X objective. Several microscope fields, equivalent to a 1 mm^2^ area, were analyzed for each rat. The empty areas within the tissue were discarded using the software Image J. A total area of 1 mm^2^ of villi and intestinal glands was analyzed per group. The identification criteria were based on the morphological characteristics of the cells, which were also quantified according to each type.

### Statistical analysis and data processing

The results were obtained from the analysis of 5 individuals per group in 2 independent experiments. The number of parasites, enteroendocrine cells, mast cells, eosinophil cells, and goblet cells located in the villi and intestinal glands were considered as dependent variables. Sleep was considered as the independent variable in two levels: sleep deprivation, and sleep sufficient. Data from 2 replicates (n = 5) from each experimental group are expressed as an average ± standard deviation, and analysed by a two way-ANOVA with Bonferroni as a *post hoc* test. Differences were considered significant when p < 0.05. The statistical data was analysed using the PRISM GraphPad software. MCA was performed as previously described 25. Briefly, MCA is a multivariate data analytic technique that provides a simple and exhaustive analysis that allows for a detailed description of the data. This analysis incorporates a bidimensional graphical output that displays clouds of points representing the categorised variables.

## Additional Information

**How to cite this article:** Ibarra-Coronado, E. G. *et al*. Innate immunity modulation in the duodenal mucosa induced by REM sleep deprivation during infection with *Trichinella spirallis.* *Sci. Rep.* **7**, 45528; doi: 10.1038/srep45528 (2017).

**Publisher's note:** Springer Nature remains neutral with regard to jurisdictional claims in published maps and institutional affiliations.
